# The impact of imported malaria by gold miners in Roraima: characterizing the spatial dynamics of autochthonous and imported malaria in an urban region of Boa Vista

**DOI:** 10.1590/0074-02760200043

**Published:** 2020-07-10

**Authors:** Jaime Louzada, Nathália Coelho Vargas de Almeida, Joao Luiz Pereira de Araujo, Júlio Silva, Thiago M Carvalho, Ananias A Escalante, Joseli Oliveira-Ferreira

**Affiliations:** 1 Universidade Federal de Roraima, Boa Vista, RR, Brasil; 2 Secretaria de Saúde de Roraima, Coordenação Geral de Vigilância em Saúde, Boa Vista, RR, Brasil; 3 Secretaria de Vigilância em Saúde do Ministério da Saúde, Brasil; 4 Fundação Oswaldo Cruz-Fiocruz, Instituto Nacional de Infectologia Evandro Chagas, Rio de Janeiro, RJ, Brasil; 5 Temple University, Institute for Genomics and Evolutionary Medicine, Department of Biology, Philadelphia, PA, United States of America; 6 Fundação Oswaldo Cruz-Fiocruz, Instituto Oswaldo Cruz, Laboratório de Imunoparasitologia, Rio de Janeiro, RJ, Brasil

**Keywords:** imported cases of malaria, spatial analysis, gold miners

## Abstract

**BACKGROUND:**

The number of malaria cases in Roraima nearly tripled from 2016 to 2018. The capital, Boa Vista, considered a low-risk area for malaria transmission, reported an increasing number of autochthonous and imported cases.

**OBJECTIVES:**

This study describes a spatial analysis on malaria cases in an urban region of Boa Vista, which sought to identify the autochthonous and imported cases and associated them with *Anopheles* habitats and the potential risk of local transmission.

**METHODS:**

In a cross-sectional study at the Polyclinic Cosme e Silva, 520 individuals were interviewed and diagnosed with malaria by microscopic examination. Using a global positional system, the locations of malaria cases by type and origin and the breeding sites of anopheline vectors were mapped and the risk of malaria transmission was evaluated by spatial point pattern analysis.

**FINDINGS:**

Malaria was detected in 57.5% of the individuals and there was a disproportionate number of imported cases (90.6%) linked to Brazilian coming from gold mining sites in Venezuela and Guyana.

**MAIN CONCLUSIONS:**

The increase in imported malaria cases circulating in the west region of Boa Vista, where there are positive breeding sites for the main vectors, may represent a potential condition for increased autochthonous malaria transmission in this space.

Malaria continues to be a significant public health problem worldwide, causing about 228 million new cases, with approximately 405,000 deaths per year, especially in Africa.^1^ Although the global incidence rate decreased by 18% between 2010 and 2016, there was a substantial increase between 2014 and 2015 in the Americas, primarily due to a surge of malaria cases in Brazil and Venezuela.^2^ In Brazil, the number of malaria cases increased from 143,748 in 2015 to 193,837 in 2018 after seven years of decline, and in Venezuela (Bolivarian Republic of Venezuela) increased from 91,918 in 2015 to 414,527 in 2017, mainly in the Bolivar State in the southeast of the country bordering with Guyana and the Brazilian State of Roraima.^3^ Also, the region has gold mining areas where working and living conditions are considered a problem for malaria control. Indeed, Bolivar State is a regional hot-spot from which malaria has spilled over to other neighboring countries overloading health care services in border municipalities of Brazil and Colombia that receive imported cases of malaria, including *Plasmodium falciparum.* ^4^

Roraima is the Brazilian State in the extreme north of the country that shares international borders with Guyana and Venezuela. In that space, human mobility across borders was usually intense but temporary. However, since 2017, Roraima has experienced the impact of an unprecedented unidirectional migration of Venezuelans due to the economic and political crisis in their country.^3^ According to UNICEF and migration authorities in Brazil, an estimate of 178,000 Venezuelans has crossed into Roraima, with at least 32,000 settlings in Boa Vista, the State capital, located approximately 100 km distant from Guyana and 200 km from Venezuela.^5^ Certainly, the increase in border population movements between Brazil, Venezuela, and Guyana have affected malaria control measures and contributed to the spread of the disease in Roraima State.^6^

Data from the Ministry of Health, SIVEP-Malaria (Epidemiologic Surveillance Information System ― Malaria), show that the number of reported cases in Roraima nearly tripled from 8,969 cases in 2016 to 23,369 in 2018.^7^ Malaria is endemic in many municipalities which have various degree of endemicity. However, 39% of the cases were imported from other states in Brazil (3,625 cases) and other countries (5,513), mainly from Venezuela (4,478 cases) and Guyana (610 cases).^10^ *P. falciparum* infections accounted for 26% of the imported and 9.8% of the autochthonous case.^7^

Boa Vista is the municipality reporting most of the malaria imported cases from neighboring countries. In 2010 the number of reported cases were 5,948 and only 6% were autochthonous.^7^ In 2011, the number of imported cases dropped to 4,354 and the autochthonous to 248 cases. This tendency to fall was maintained until reaching its lowest value in 2016, with about 2,357 imported cases and 12 autochthonous. However, in the years 2017 to 2018 there was a new increase in imported cases.^7^

In 2018, Boa Vista reported 5,713 cases, 168 (3%) autochthonous, 2,805 (49%) imported from other municipalities/states in Brazil, and 2,740 (48%) from other countries, mainly Venezuela (n = 2,115) and Guyana (n = 533). It is also noteworthy that, in 2018, cases of *P. falciparum* infection from Venezuela (n = 481) and Guyana (n = 97) represent 80% of the notifications of *P. falciparum* registered in Boa Vista (722 cases).^7^ Studies of malaria vectors in Roraima performed more than ten years ago found that *Anopheles albitarsis* and *An. darlingi* were infected with *Plasmodium* spp.^8^ ^,^ ^9^

Despite being considered a low-risk area for malaria transmission, the consistent numbers of imported malaria cases, which are a possible source of infection, together with the presence of competent vectors in urban areas, make possible outbreaks and epidemics in Boa Vista.^10^ A sustained increase in local transmission would be a significant setback for malaria elimination efforts in Boa Vista.

Considering the multiple factors that may drive the increase in malaria observed in Brazil, disentangling their differential contribution will guide how to better deploy interventions. Therefore, this study aimed to understand the epidemiology of the imported malaria cases and the potential risk of increased transmission in an urban region of Boa Vista.

## SUBJECTS AND METHODS

**Fig. 1:**
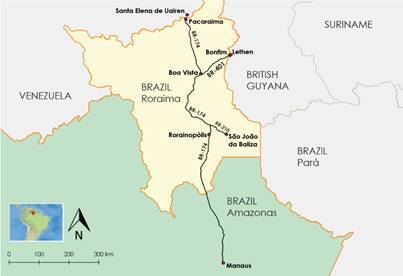
maps of Brazil and Roraima State in the border of Venezuela and Guyana. The studied site Boa Vista and the border municipalities of Pacaraima and Bonfim. Source: by the authors themselves.

*Study site* - The study was carried out in Boa Vista, Roraima State, the only Brazilian State capital located entirely in the northern hemisphere, between Latitude 02º49’12” N and Longitude 60º40’23”W. Boa Vista occupies an area of 5,687,037 Km^2^, concentrates 63.4% of the state’s population and has a demographic density of 49.99 inhabitants per Km^2^ (Instituto Brasileiro de Geografia e Estatística - IBGE, 2016). Savanna is the predominant ecological environment in Boa Vista and the climate presents two distinct seasons: a rainy season between April and November with high rainfall indices during the months of June and July and the dry season between December and March. The geographical position of the municipality allows easy access to international borders thus positioned: in the north part of the state in the municipality of Pacaraima, which borders Santa Elena do Uairén in Venezuela, 231.5 km from Boa Vista, a land route (BR- 174). The municipality of Bonfim bordering Lethem, Cooperative Republic of Guyana, 133.3 km from Boa Vista via BR-401. To the South, the State is bordered by Amazonas State and Pará, Fig. 1.

*Study population and sample collection* - Samples and research data were collected from March 2016 to September 2018 at the Polyclinic Cosme e Silva. It is a health unit of the Unified Health System (SUS) that has a high demand for various medical specialties. This Polyclinic is strategically located in the west area of the city of Boa Vista, which has a population of 152,062 inhabitants (IBGE - 2016), distributed in 33 (thirty-three) neighborhoods. According to data from Ministry of Health, SIVEP - Malaria, among the 13 health units that offer malaria diagnosis in Boa Vista, the Polyclinic was the one that registered the highest number of cases in the last 5 years, as follow: in 2011, 2,354 cases; in 2012, 1,424 cases; in 2013, 1,713 cases; in 2014, 1,384 cases; and 966 cases in 2015.^10^ Taking in account the total number of reported cases in Boa Vista in the same period (2011-2015), the Polyclinic Cosme e Silva was responsible for 51%, 44%, 42%, 54% and 48.8% of the reported cases, respectively.^7^ Therefore, all individuals seeking a malaria diagnosis in the Public Health Unit Polyclinic Cosme e Silva, were eligible to participate in the study.

A questionnaire, completed by each participant, was used to collect demographic data, travel history, and epidemiological data. Blood was drawn from each participant by venipuncture and malaria infection was diagnosed by microscopic examination of Giemsa stained blood smears. The parasitological evaluations were performed by examination of 200 fields at 1,000-fold magnification under oil immersion. Parasite densities were estimated by counting the number of parasites per microliter of blood (all species and stages) per 200 leukocytes in thick blood films, multiplying this by the number of individual leukocytes, and dividing this by 200.

All positive individuals were treated with antimalarial drugs, according to the protocol of the Brazilian Ministry of Health. All the questionnaire data were stored in Epi Info™ 2002 (CDC, Atlanta, GA, USA). This protocol was approved by the Federal University of Roraima Ethical Committee (CAAE: 44055315.0.0000.5302).

*Case definition* - All malaria cases were classified as autochthonous or imported depending on the most likely place of acquisition as per the questionnaire. In this study, imported malaria was defined as malaria acquired outside the municipality of Boa Vista but diagnosed within the municipality. This was determined by the individual history of staying in an endemic municipality, state or country during the last one month. Autochthonous cases were defined as malaria acquired in the municipality of Boa Vista in persons without travel history outside the municipality in the last one month.

*Breeding sites and larval collection* - Twenty-three breeding sites were identified and georeferenced using Garmin GPS by technicians of the entomology service of the General Coordination of Health Surveillance of Roraima. The breeding sites were chosen based on the location of autochthonous cases in previous 3 years (2014 to 2016). These breeding sites have been monitored by the entomology service of the General Coordination of Health Surveillance every two months between 2017 and 2018 and only qualitatively classified as positive or negative for the presence of *Anopheles* spp larvae. The larvae collections were performed using standard entomological scoops (BioQuip, Ranch Dominguez, CA, USA). At each breeding site, nine scopes were made, three to the right, three to the front, and three to the left, following the Ministry of Health standard.^10^ Due to the great difficulty in rearing *Anopheles* larvae stage in the laboratory, *Anopheles* larvae from 1st and 2nd was used to determine if the breeding place were positive for *Anopheles* spp. Larvae (third and fourth-instar) and adults were identified using the keys of Consoli and Lourenço-de-Oliveira and Forattini.^11^ ^,^ ^12^ The larvae collected were transferred to plastic bags, labeled with the breeding site code, date and collector. All collected material was transferred to the state entomology laboratory to be screened and identified. The breeding site was considered positive if in at least one collection per year presented *Anopheles* spp larvae.

*Spatial analysis* - The spatial dynamics of malaria in Boa Vista were analysed considering three situations: mapping of individuals with malaria, positive breeding sites for *Anopheles* and circulation time between first symptoms and treatment initiation. To perform spatial analysis, the locations of disease occurrence was recorded based on the address of malaria patients (Street and Neighborhood) and geographic coordinates were obtained, in decimal degrees geographic coordinate system, via Google Maps geographic location service.

The data were processed in the QGIS 2.14.17 software, executed from the Windows platform, to generate maps.

The basic cartography set was georeferenced using the decimal degrees geographic coordinate system and the World Geodetic System-1984 reference system (WGS84). All geographic data were processed in the Landscape Metrics Laboratory of the Department of Geography of the Federal University of Roraima (UFRR), which has available satellite images and cartographic basis of Boa Vista for the preparation of thematic maps.

*Statistical analysis and spatial point pattern analysis* - Demographic, epidemiological and parasitological data were entered into a database created using the Epi Info software (Epi Info™, Atlanta, GA, USA). For statistical purpose, the following variables were taken into consideration: gender, age, residence, nationality, occupation, place of infection, symptoms, days since first symptoms and past and present malaria infections. Proportions were compared using 2x2 contingency tables with either chi-squared tests, adjusted by Yates’ continuity correction or Fishers exact tests, as appropriate. Odds ratios (OR) analysis were used to quantify the strength of the association between variables. The statistical significance threshold was p < 0.05, with 95% confidence intervals (95% CI) for all hypothesis tests. These analyses were performed using GraphPad InStat, version 3 (GraphPad Software, San Diego, CA, USA).

Kolmogorov-Smirnov test was used to verify the hypothesis of risk for malaria transmission in the western region of Boa Vista due to the existence of breeding sites and individuals with imported malaria, part of which with gametocytes. This test was applied in order to assess whether the distribution of the points follows a homogeneous Poisson process or whether the points are distributed according to a clustering model. For the interpretation of the values, consider a 5% level of significance in the results (95% CI). In summary, the null hypothesis to be tested is that the distribution of points throughout the region under study follows a homogeneous Poisson process.^13^ ^,^ ^14^

In order to verify the hypothesis of complete spatial randomness, the analysis of specific processes was carried out by evaluating the graph of the G, F, K and L functions, to verify whether the points are distributed randomly or in the form of grouping throughout the region under study. The G function represents the distance from the nearest event (breeding sites, malaria and gametocytes cases), the plotted results of this function can be used as an exploratory method to check if there is evidence of interaction between the events; F function aims to check the risk rate, since it measures the distribution of all distances from an arbitrary point (cases, breeding sites and gametocytes) to the point closest to it; the K function represents the Ripley’s function, that allows the detection of the spatial pattern at different distance scales simultaneously; while the L function consists of the Besag transformation function of the Ripley’s function.^15^ In the horizontal axis of the graphs it is shown distances between the points (individuals with malaria, breeding sites and gametocytes), so that in the vertical axis we have the density values based on the estimators of the functions G, F, K and L, respectively. The black lines mean the observed values, while the red lines correspond to the theoretical values of the function. The gray value range consists of the 95% CI, considering 99 simulations. The analyzes were performed using the software R v. 3.6.2.

## RESULTS

*Characteristic of the studied population* - During the study period, 520 individuals participated in the study, 299 (57.5%) were positive for *Plasmodium* in the thick blood smear. The characteristics of the study population are presented in Table I. The majority of the individuals were male (74.6%), the mean age was 36 years and the nationality was predominantly Brazilians with permanent residence in Boa Vista, Roraima State. However, their main occupation was gold mining or mining activities in Venezuela and Guyana. Overall, 468 individuals declared a history of malaria episodes (90%); the majority reported more than five episodes (74.4%) and 10% did not recall past infection in their lifetime. Ninety-six percent of the asymptomatic individuals (n = 19), 68.4% had a positive diagnosis for malaria while 31.6% were negative. No difference was observed in the nationality, place of residence, occupation, and number of malaria episodes in the last three years between positive and negative individuals. However, proportion of malaria positives individuals decreases in the age groups 36-44 (OR = 0.59, 95% CI 0.35 - 0.98; p = 0.04) and > 44 (OR = 0.36, 95% CI 0.21 - 0.6; p < 0.01), as compared to groups of younger ages.

**TABLE I:** Characteristics of the study population according to malaria diagnosis in Boa Vista, Roraima State during 2016 to 2018

Variables	Malaria diagnosis			Odds ratios (95% CI)	p-value
	Positive 299	Negative 221	Total 520				
Gender n (%)					
Male	225 (75.2)	163 (73.7)	388 (74.6)	1.08 (0.73-1.61)	0.7
Female	74 (24.8)	58 (26.3)	132 (25.4)	1	-
Age n (%)					
< 28	92 (30.8)	41 (18.5)	133 (25.6)	1	-
28 - 35	83 (27.7)	60 (27.1)	143 (27.5)	0.62 (0.38-1.01)	0.06
36 - 44	70 (23.4)	53 (24.0)	123 (23.6)	0.59 (0.35-0.98)	0.04
> 44	54 (18.1)	67 (30.4)	121 (23.3)	0.36 (0.21-0.6)	< 0.01
Nationality n (%)					
Brazilian	282 (94.3)	215 (97.3)	497 (95.6)	1	-
Venezuelan	15 (5.0)	6 (2.7)	21 (4.0)	1.9 (0.72-4.98)	0.19
Guyanese	2 (0.7)	0 (0)	2 (0.4)	-	-
Occupation n (%)					
Gold mining	186 (62.2)	150 (67.9)	336 (64.6)	1	-
Other occupation	113 (37.8)	71 (32.1)	184 (35.4)	1.28 (0.89-1.85)	0.18
Residence n (%)					
Boa Vista	293 (98.0)	218 (98.7)	511 (98.2)	-	-
Other municipalities/state	6 (2.0)	3 (1.3)	9 (1.8)	1.49 (0.37-6.02)	0.58
Past ME n (%)					
> 5	222 (74.2)	165 (74.7)	387 (74.4)	1	-
≥ 5	40 (13.4)	41 (18.6)	81 (15.6)	0.8 (0.5-1.29)	0.37
Never had	37 (12.4)	15 (6.7)	52 (10.0)	1.72 (0.87-3.39)	0.12
ME last 3 years median (IQR)	0 (0.1)	1 (0.1)	1 (0.1)	1.1 (0.88-1.38)	0.41
Days since last ME median (IQR)	60 (30.180)	60 (30.180)	60 (30.180)	1 (1.1)	0.76
Symptoms n(%)					
Yes	286 (95.6)	215 (97.2)	501 (96.3)	1.36 (0.43-4.28)	0.6
No	13 (4.4)	6 (2.8)	19 (3.7)	1	-

m ± sd: mean ± standard deviation; ME: malaria episodes. Odds ratios analysis were used to quantify the strength of the association between variables. P value < 0.05 were significative. IQR: interquartile range.

*Breeding sites* - Boa Vista is located in an area with large rivers, streams and lakes, characteristics that favors the existence of breeding sites, in addition to the climate favorable to the presence of the main malaria vectors. The breeding sites were lakes, streams and natural permanent rivers, partially shaded and sunny with the presence of marginal vegetation, mainly in the dark and still waters. All 23 breeding sites mapped mostly murky and standing waters. All 23 were larval habitats for *Anopheles* spp larvae. *An. darlingi* and *An. albitarsis* were the species found in all investigated breeding sites, however, other species were as also found, *An*. *Nuneztovari* s.l., *An*. *Mattogrossensis*, *An*. *Triannulatus* and *An*. *Evansae.*

*Autochthonous and imported malaria cases* - Of the 299 individuals positive for malaria infection during the studied period, 294 (98.3%) were imported and only five (1.7%) were autochthonous from the municipality of Boa Vista. Among these 172 were infected with *P. vivax*, 109 with *P. falciparum*, and 13 with mixed (*P. falciparum* + *P. vivax*). *P. vivax* was the most prevalent *Plasmodium* species found in 100% of the autochthonous cases and 57.5% of the imported cases. The observed *P. falciparum* and mixed (*P. falciparum* + *P. vivax*) infections were imported, Table II.

Brazilian were the majority of the cases (94.6%) followed by Venezuelan (5.1%) and Guyanese (0.3%). A large number of the imported cases were acquired in Venezuela (58.8%) and Guyana (32.3%). The cases from French Guyana and Suriname corresponded to 1%) and 7.7% came from other municipalities of Roraima or other states in Brazil. Individuals with *P. falciparum* had, on average, a greater parasitemia than individuals affected by *P. vivax* and mixed infections. On average patients infected with *P. falciparum* and/or mixed infections sought diagnosis after about five days of onset of symptoms.

**TABLE II:** Characteristics of imported and autochthonous malaria infection diagnosed in Boa Vista, Roraima State during 2016 to 2018

Variables	Autochthonous	Imported	Total
Malaria infection n (%)	5 (1.7)	294 (98.3)	299 (57.5)
Age n(%)			
< 28	3 (60)	89 (30.3)	92 (30.8)
> 44	0 (0)	54 (18.4)	54 (18.1)
28-35	1 (20)	82 (27.9)	83 (27.8)
36-44	1 (20)	69 (23.5)	70 (23.4)
Gender, Male n (%)	4 (80)	221 (75.2)	225 (75.3)
*Plasmodium* species n (%)			
*P. falciparum*	0 (0)	109 (37.1)	109 (36.5)
*P. vivax*	5 (100)	172 (58.5)	177 (59.2)
*P. falciparum* + *P. vivax*	0 (0)	13 (4.4)	13 (4.3)
Nationality n (%)			
Brazilian	5 (100)	278 (94.6)	283 (94.6)
Guyanese	0 (0)	1 (0.3)	1 (0.3)
Venezuelan	0 (0)	15 (5.1)	15 (5)
Place of infection n (%)			
Boa Vista	5 (100)	0 (0)	5 (1.7)
Guyana	0 (0)	95 (32.3)	95 (31.8)
Other countries	0 (0)	3 (1)	3 (1)
Other municipalities/states	0 (0)	23 (7.8)	23 (7.7)
Venezuela	0 (0)	173 (58.8)	173 (57.9)
Parasitemia median (IQR)			
*P. falciparum*	-	503 (280,900)	503 (280,900)
*P. vivax*	800 (463,1200)	462 (287.2,900.8)	468 (298,903)
*P. falciparum* + *P. vivax*	-	534 (410,761)	534 (410,761)
Days since first symptoms median (IQR)	6 (6,13)	4 (3,6)	4 (3,6)

m ± sd: means ± standard deviation; days since first symptoms: time interval between first symptoms and diagnosis and treatment. IQR: interquartile range.

**Fig. 2:**
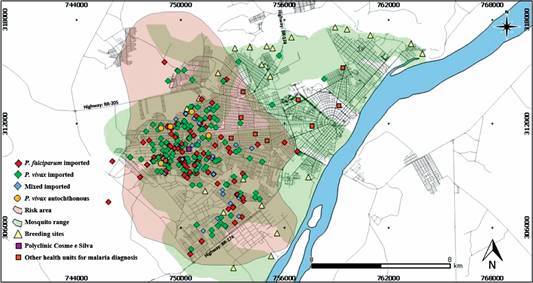
distribution of malaria cases by species, origin and breeding sites positive for *Anopheles* spp immature forms in urban environment of Boa Vista.

*Spatial distribution of infecting malaria species and vectors* - The available addresses of 271 imported and five autochthonous cases were used to plot the spatial distribution of the cases by species, Fig. 2. Only two out of five autochthonous *P. vivax* cases reported their residence; the other three reported the place of infection as the neighborhood where they work. All individuals reported not leaving the municipality in the last 1 month.

A radius of 1 km was established from the identified breeding sites as the mosquito’s flight coverage area (green region of the map).^16^ ^,^ ^17^ An area at risk of malaria transmission was delimited on the map (pink region of the map) considering the location of autochthonous and imported cases and their spatial relations with the breeding sites. In Fig. 2, the majority plotted points were of *P. vivax* malaria (151 imported and five autochthonous), *P. falciparum* malaria corresponds to 88 georeferenced points, while 32 points on the map represent mixed malaria. The map includes the 23 georeferenced breeding sites in the city. We observed that the majority of cases were concentrated in the west of the city. Several breeding sites were also observed in the east side of the city (green area in Fig. 2).

**Fig. 3:**
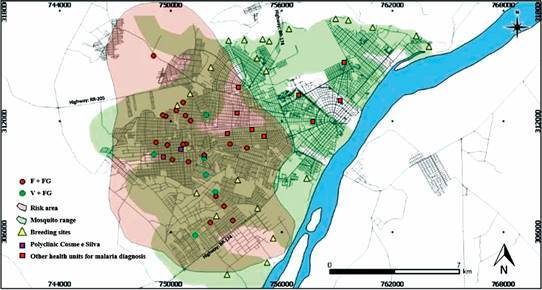
distribution of gametocytes and vectors in the urban environment of Boa vista. F+FG: individuals with thick blood smear positive for *Plasmodium falciparum* blood stages including *P. falciparum* gametocytes; V+FG: individuals with positive thick blood smear for *P. vivax* blood stages including *P. vivax* gametocytes.

*Spatial distribution of gametocytes carriers and breeding sites* - The residence of individuals positive for gametocyte forms of *P. falciparum* and *P. vivax* and their relationship with breeding sites was plotted, Fig. 3. We observed that gametocyte positive individuals were found within the radius of action of the *Anopheles* established at 1 km (green area) from the larval collection points in the streams and rivers positive for both *An. darlingi* and *An. albitarsis*. The highest concentration of these individuals was in the south and west of the municipality. The latter has the largest number of neighborhoods, 33, and it is the most populous with 152,062 inhabitants. Most of these neighborhoods are in the city outskirts and are within the breeding places range (pink color on the map of Fig. 3). Noteworthy, 73% of the gametocyte’s carriers sought treatment 48 h after the symptoms had started and these individuals were living in areas at risk of malaria transmission (pink color on the map) in several districts of Boa Vista, including the most populous zone of the municipality.

*Risk of local malaria transmission* - According to the test results [Kolmogorov-Smirnov = 0.2376 (p-value < 0.001 in 95% CI)], there are specific locations that tend to have a higher frequency of malaria cases. It is to say that the circulation of individuals with malaria along the west zone of the municipality of Boa Vista increases the risk of malaria transmission.

**Fig. 4:**
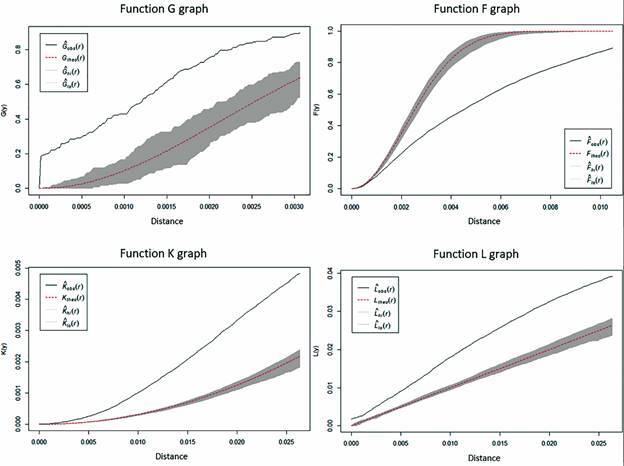
analysis of the specific process functions G, F, K, L. Function: G (Distance function from the nearest event); Function: F (Risk rate function); Function: K (Ripley function); Function: L (Besag transformation function of Ripley function). Horizontal axis of the graphs: distances between the points (individuals with malaria, breeding sites and gametocytes); vertical axis: density values based on the estimators of the functions G, F, K and L, respectively. Black lines: observed values; red lines: theoretical values of the function; gray value range: 95% confidence interval, considering 99 simulations.

Results in Fig. 4 (G function) show that the observed values, that is, the distance between the cases of malaria and individuals carrying gametocytes from breeding sites (black line), are outside and above the confidence interval given by the gray area. Therefore, the circulation of individuals with the disease close to breeding sites is a risk factor for local malaria transmission. Analysis in a wider area, Fig. 4 (function F), the observed values, that is, the risk rate (black line), are outside and below the confidence interval given by the gray area. Thus, by the behavior of the function graph, the presence of individuals with malaria in this part of the municipality poses risk of local malaria transmission. Indeed, in Fig. 4. The reduced second order measures (K and L functions), endorse risk of local malaria transmission indicated in function G and K. Therefore, the spatial distribution and proximity of malaria cases, individuals carrying gametocytes and malaria vector breeding sites favors local malaria transmission.

## DISCUSSION

Roraima State has suffered an increase in the number of malaria cases. Its capital, Boa Vista, is the municipality with the highest number of imported cases in the State.^3^ Imported malaria is considered a critical impediment to elimination in many countries including Brazil. Indeed, this study shows that most of the cases reported in the Health Unit in the urban area of Boa Vista, during the time of this study, were imported from Venezuela (58.8%) and Guyana (32.3%) with only few autochthonous cases (1.7%), as previously observed.^7^ Autochthonous infections were all *P. vivax* while imported infections were attributed mainly to *P. vivax* and to a lesser extent to *P. falciparum*. Despite its relatively low number, *falciparum/vivax* mixed infections were detected in the study’s site. Moreover, the vast majority of these imported cases (90.4%) were Brazilians coming from gold mining sites (90.4%) in Venezuela and Guyana rather than migrants from these countries. Overall, gold miners are at higher risk of contracting malaria making them a “hot pop” or subpopulation at higher risk of *Plasmodium* infection.^18^

Brazilian miners spend part of their time between the gold mines abroad and Boa Vista, where they live (98.3%) and sell the products of this labor, generating high mobility between endemic areas in Venezuela and Guyana to Boa Vista. Indeed, the massive flow of miners in this health unit reflects the high risk of malaria in the mining sites.^7^ ^,^ ^19^ Consistent with these observations, imported malaria in recent years has increased exponentially as gold mining activities increase in 2018 and gold was the second most exported product in the state.^19^ ^,^ ^20^ ^,^ ^21^ There are currently 1,097 illegal mining points located in Venezuela, Guyana and Brazil (Yanomami indigenous lands) producing high mobility of job seekers.^22^ Although these mining regions are generally unhealthy and dangerous, it provides an economic incentive that motivates the return of these individuals in search of survival, while still at risk of malaria. Overall, the situation observed in Roraima is consistent with reports emerging from other endemic areas in the Americas.^23^ ^,^ ^24^

Results from our study indicate that there are several positive breeding sites for *An. darlingi and An. albitarsis* in Boa Vista. Also, we found asymptomatic individuals (4.3%) and the “time to start treatment of patients after the date of first symptoms” was over 48 h for 60% of patients. These factors could increase the risk of local transmission after malaria has been introduced. This situation requires increasing surveillance as evidenced by an increase of 1.300% in autochthonous cases in Boa Vista in 2018.

Importantly, the Boa Vista health services attract migrants from other municipalities and border cities seeking malaria treatment and the location of the city favors the movement of people and goods. These characteristics create a continuous flow between the mining areas and Boa Vista. The relationship between people’s mobility and imported malaria has been pointed out in several studies as a concern for malaria control and elimination. In this case, mobility is linked to gold miners.

The risk of local malaria transmission observed by the spatial point pattern analysis, even if it is limited to an area of Boa Vista, deserves attention from health surveillance agencies, as it is a populous area where most neighborhoods are concentrated, in addition to housing farms and water collection. It is possible that this risk may extend to other areas of the city given the similarity of the characteristics observed in the urban and peri-urban perimeter of the municipality. Therefore, the expansion of this study to other areas of the city is important in order to assess this risk for the city of Boa Vista as a whole.

In Boa Vista, during the study period, the main malaria control measures were passive case detection and treatment and other measures follow a specific demand focused on the occurrence of autochthonous cases. Active case detection of infected individual’s, insecticide spraying of households and health education are limited to the probable place of infection of confirmed autochthonous case and neighborhood. Occasionally, identification of breeding sites and *Anopheles* larvae collection are performed. Therefore, implementation of control measures to reduce the risk of autochthonous transmission such as continuous surveillance of active case detection, monitoring *Anopheles* breeding sites associated with insecticide spraying of households and health education targeting the hot pop, especially gold miners should be strongly considered in Boa Vista.^25^ ^,^ ^26^

It is worth noting that we did not observe any autochthonous *P. falciparum* infections, although our number was low (n = 5). However, data from the Ministry of Health, SIVEP-Malaria have indicated 14 *P. falciparum* imported cases in Boa Vista in the last three years.^7^ Imported *P. falciparum* cases present an unusual distribution in other studies, affecting about two out of five individuals examined, a cause for concern as there is a risk of introducing and spreading *P. falciparum* resistant strains from neighboring countries in Brazil.^27^ ^,^ ^28^ In a macro-policy scenario, advocated by the Ministry of Health, through the elimination plan, Brazil aims to eliminate *falciparum* malaria by 2022.^28^

There is no bilateral cooperation between Brazil and Venezuela such as that between Andean countries (Venezuela, Peru, Colombia, and Ecuador) for malaria containment.^28^ However, since Brazilians miners are the ones introducing malaria, this may reveal weaknesses in border control measures, and poses a challenge for malaria control and, particularly, for *P. falciparum* elimination goals. Given the relatively good communication between Bolivar State and Roraima, and the gold mining activities in Bolivar State, the Brazilian-Venezuelan border could be regarded as a malaria corridor where the disease will likely remain resilient to uncoordinated control efforts across international borders.^29^

The data reported in this research are important, however limited, as they represent only a certain area of the city of Boa Vista. In this study, participation of Brazilians with malaria was significantly higher than Venezuelans, with the majority of imported malaria coming from the mining region. As this study was limited to just one health unit, it is possible that other health centers provided assistance to Venezuelans with malaria.

Despite this limitation, it is important to note that in regions with low malaria transmission, the distribution of autochthonous cases has become more and more irregular and heterogeneous, capable of modifying local epidemiology, as observed by the increase of autochthonous cases in Boa Vista (1.300%).

*In conclusion* - Imported malaria in an urban area of Boa Vista is driven by Brazilians working on gold mines traveling across international borders. It seems that the origin of imported malaria in the west region of Boa Vista is related to mining and not to the migratory movement. Regardless of the motivation, malaria represents the universe of individuals at social risk. Despite the low number of autochthonous cases, the influx of imported infected travelers into areas with suitable vectors breeding sites, there is a potential risk for an increase in autochthonous malaria in the studied area, including the risk of introducing *P. falciparum* malaria. Therefore, early detection of malaria cases is necessary to limit their potential negative impact in achieving the goals of elimination plan proposed by the Brazilian Ministry of Health.

## References

[1] WHO - World Health Organization (2019). World Malaria Report 2019.

[2] Espinoza JL (2019). Malaria resurgence in the Americas an underestimated threat. Pathogens.

[3] Grillet ME, Hernandez-Villena JV, Llewellyn MS, Paniz-Mondolfi AE, Tami A, Vincenti-Gonzalez MF (2019). Venezuela's humanitarian crisis, resurgence of vector-borne diseases, and implications for spillover in the region. Lancet Infect Dis.

[4] Daniels JP (2018). Increasing malaria in Venezuela threatens regional progress. Lancet Infect Dis.

[5] Unicef (2019). Crise migratória venezuelana no Brasil. https://www.unicef.org/brazil/crise-migratoria-venezuelana-no-brasil..

[6] Wangdi K, Gatton ML, Kelly GC, Clements AC (2015). Cross-border malaria a major obstacle for malaria elimination. Adv Parasitol.

[7] Ministério da Saúde (2020). htpp//portalweb04.saude.gov.br/Sivep_malaria/.

[8] Póvoa MM, de Souza RTL, Lacerda RNL, Rosa ES, Galiza D, de Souza JR (2006). The importance of Anopheles albitarsis E and An darlingi in human malaria transmission in Boa Vista, state of Roraima, Brazil. Mem Inst Oswaldo Cruz.

[9] Rosa-Freitas MG, Tsouris P, Peterson AT, Honorio NA, de Barros FSM, de Aguiar DB (2007). An ecoregional classification for the state of Roraima, Brazil The importance of landscape in malaria biology. Mem Inst Oswaldo Cruz.

[10] Secretaria de Vigilância em Saúde, Ministério da Saúde (2011). Padronização dos métodos utilizados em pesquisa larvária de Anopheles na rotina dos laboratórios de entomologia.

[11] Consoli RAGB, Lourenço-de-Oliveira R (1994). Principais mosquitos de importância sanitária no Brasil.

[12] Forattini OP (2002). Culicidologia médica. Vol. 2: Identificação, Biologia, Epidemiologia. Edusp.

[13] Durbin J (1971). Boundary-crossing probabilities for the Brownian motion and Poisson processes and techniques for computing the power of the Kolmogorov-Smirnov test. J Appl Probab.

[14] Câmara G, Carvalho MS, Cruz OG, Correia V, Druck S, Carvalho MS, Câmara G, Monteiro AMV (2004). Análise espacial de áreas. Análise espacial de dados geográficos.

[15] Lewis PAW (1965). Some results on tests for Poisson processes. Biometrika.

[16] Guerra CA, Reiner RC, Perkins TA, Lindsay SW, Midega JT, Brady OJ (2014). A global assembly of adult female mosquito mark-release-recapture data to inform the control of mosquito-borne pathogens. Parasit Vectors.

[17] Charlwood JD, Alecrim WA (1989). Capture-recapture studies with the South American malaria vector Anopheles darlingi, Root. Ann Trop Med Parasitol.

[18] Cotter C, Sturrock HJ, Hsiang MS, Liu J, Phillips AA, Hwang J (2013). The changing epidemiology of malaria elimination new strategies for new challenges. Lancet.

[19] Douine M, Mosnier E, Le Hingrat Q, Charpentier C, Corlin F, Hureau L (2017). Illegal gold miners in French Guiana a neglected population with poor health. BMC Public Health.

[20] Fellet J (2019). Roraima exporta 194 kg de ouro à Índia sem ter nenhuma mina operando legalmente.. BBC News Brasil.

[21] Douine M, Musset L, Corlin F, Pelleau S, Pasquier J, Mutricy L (2016). Prevalence of Plasmodium spp. in illegal gold miners in French Guiana in 2015: a hidden but critical malaria reservoir. Malar J.

[22] Rede Amazônica de Informação Socioambiental Georeferenciada https://www.amazoniasocioambiental.org/pt-br/.

[23] Castellanos A, Chaparro-Narváez P, Morales-Plaza CD, Alzate A, Padilla J, Arévalo M (2016). Malaria in gold-mining areas in Colombia. Mem Inst Oswaldo Cruz.

[24] Musset L, Pelleau S, Girod R, Ardillon V, Carvalho L, Dusfour I (2014). Malaria on the Guiana Shield a review of the situation in French Guiana. Mem Inst Oswaldo Cruz.

[25] Xu C, Wei QK, Li J, Xiao T, Yin K, Zhao CL (2016). Characteristics of imported malaria and species of Plasmodium involved in Shandong province, China (2012-2014). Korean J Parasitol.

[26] Sriwichai P, Karl S, Samung Y, Kiattibutr K, Sirichaisinthop J, Mueller I (2017). Imported Plasmodium falciparum and locally transmitted Plasmodium vivax cross-border malaria transmission scenario in northwestern Thailand. Malar J.

[27] Chenet SM, Akinyi Okoth S, Huber CS, Chandrabose J, Lucchi NW, Talundzic E (2016). Independent emergence of the Plasmodium falciparum Kelch propeller domain mutant allele C580Y in Guyana. J Infect Dis.

[28] Ferreira MU, Castro MC (2016). Challenges for malaria elimination in Brazil. Malar J.

[29] Recht J, Siqueira AM, Monteiro WM, Herrera SM, Herrera S, Lacerda MVG (2017). Malaria in Brazil, Colombia, Peru and Venezuela current challenges in malaria control and elimination. Malar J.

